# Time-Resolved Transcriptomics and Constraint-Based Modeling Identify System-Level Metabolic Features and Overexpression Targets to Increase Spiramycin Production in *Streptomyces ambofaciens*

**DOI:** 10.3389/fmicb.2017.00835

**Published:** 2017-05-12

**Authors:** Marco Fondi, Eva Pinatel, Adelfia Talà, Fabrizio Damiano, Clarissa Consolandi, Benedetta Mattorre, Daniela Fico, Mariangela Testini, Giuseppe E. De Benedetto, Luisa Siculella, Gianluca De Bellis, Pietro Alifano, Clelia Peano

**Affiliations:** ^1^Department of Biology, University of FlorenceFlorence, Italy; ^2^Institute of Biomedical Technologies, National Research CouncilSegrate, Italy; ^3^Department of Biological and Environmental Sciences and Technologies, University of SalentoLecce, Italy; ^4^Laboratory of Analytical and Isotopic Mass Spectrometry, Department of Cultural Heritage, University of SalentoLecce, Italy

**Keywords:** *Streptomyces ambofaciens*, antibiotic production, strain improvement, metabolic modeling, transcriptomics, systems biology

## Abstract

In this study we have applied an integrated system biology approach to characterize the metabolic landscape of *Streptomyces ambofaciens* and to identify a list of potential metabolic engineering targets for the overproduction of the secondary metabolites in this microorganism. We focused on an often overlooked growth period (i.e., post-first rapid growth phase) and, by integrating constraint-based metabolic modeling with time resolved RNA-seq data, we depicted the main effects of changes in gene expression on the overall metabolic reprogramming occurring in *S. ambofaciens*. Moreover, through metabolic modeling, we unraveled a set of candidate overexpression gene targets hypothetically leading to spiramycin overproduction. Model predictions were experimentally validated by genetic manipulation of the recently described ethylmalonyl-CoA metabolic node, providing evidence that spiramycin productivity may be increased by enhancing the carbon flow through this pathway. The goal was achieved by over-expressing the *ccr* paralog *srm4* in an *ad hoc* engineered plasmid. This work embeds the first metabolic reconstruction of *S. ambofaciens* and the successful experimental validation of model predictions and demonstrates the validity and the importance of *in silico* modeling tools for the overproduction of molecules with a biotechnological interest. Finally, the proposed metabolic reconstruction, which includes manually refined pathways for several secondary metabolites with antimicrobial activity, represents a solid platform for the future exploitation of *S. ambofaciens* biotechnological potential.

## Introduction

Constraint-based modeling is a widely adopted technique to study microbial metabolic features at the system level. Indeed, it has proven useful for addressing fundamental issues about the metabolic landscape of a variety of microorganisms, including the elucidation of non-trivial metabolic engineering strategies (King et al., 2015; Zhang and Hua, 2016). Genome-scale metabolic networks are formulated and reconstructed using genomic information of the species to be modeled. Next, computational methods (e.g., Flux Balance Analysis, FBA) can be adopted to infer the flux distributions within the cell (Orth et al., 2010), and, even more importantly, to study the metabolic reprogramming following environmental perturbations and/or changes in any of the possible cellular information layers (e.g., gene expression). Many fields can benefit from this technology including clinical microbiology (Sigurdsson et al., 2012), metabolic engineering (Licona-Cassani et al., 2012), environmental microbiology (Fondi et al., 2015), and microbial ecology (Stolyar et al., 2007). A relevant (recent) achievement in the field of constraint-based modeling is the possibility to integrate gene expression data into the metabolic framework. Indeed, by constraining the admissible flux across each reaction in the model on the basis of the corresponding genes’ expression level, metabolic flux predictions can be improved or context-specific models created. Despite the fact that a recent work showed that no method performs universally well, the integration of transcriptomics data and constraint-based metabolic modeling might still provide cues to guide the determination of the correct phenotype among the space of solutions (Machado and Herrgard, 2014).

*Streptomyces* representatives’ metabolism has been extensively studied (Baltz, 2016), in many cases using genome-scale metabolic reconstruction and constraint-based modeling (Borodina et al., 2005; D’Huys et al., 2012; Huang et al., 2012, 2013). In particular, this computational approach has disclosed potential metabolic engineering strategies (e.g., overexpression and knock-out targets) for daptomycin and FK506 overproduction in *S. roseosporus* and *S. tsukubaensis*, respectively. Furthermore, a comprehensive and system-level understanding of the metabolic landscape of *S. lividans* and *S. coelicolor* has been depicted using this methodology, to predict flux changes that occur when the cell switches from biomass to antibiotic production (Alam et al., 2010). Interestingly, the potential of integrating gene expression data and metabolic modeling for exploring *Streptomyces* biology (and biotechnological potential) has been assessed in a recent work focused on *S. coelicolor* (Kim et al., 2016). Besides benchmarking currently available tools for transcriptomic data integration into metabolic reconstructions and identifying the outperforming tool among them (iMAT, Shlomi et al., 2008), this study has identified a list of potential metabolic engineering targets for the overproduction of the actinorhodin secondary metabolite in this and other streptomycetes.

Our study offers a system biology approach to explore, from a global point of view, the metabolic landscape of *Streptomyces ambofaciens*, a prolific producer of bioactive compounds. The reference strain *S. ambofaciens* ATCC 23877 (Pinnert-Sindico, 1954), whose genome sequence has been recently delivered (Thibessard et al., 2015), has been known for the last 60 years due to its ability to produce a wide range of secondary metabolites including spiramycin, a macrolide used in human medicine as antibacterial and antiparasitic agents (active against *Toxoplasma* spp.) (Poulet et al., 2005; Chew et al., 2012) and congocidine (netropsin), a pyrrolamide with a broad range of biological activities but no medical applications (Cosar et al., 1952). More recently, a genome mining-guided approach has revealed the ability of this strain to produce, in addition to spiramycin and congocidine, the antibiotic kinamycin, the siderophores coelichelin and desferioxamines, the antifungals antimycin and stambomycins, and novel polyketides with antibacterial and antiproliferative activities (Thibessard et al., 2015).

Here we have applied an approach that combines physiological, transcriptomic and *in silico* modeling with the aim to provide a system-level understanding of *S. ambofaciens* metabolic features, and to identify a list of potential metabolic engineering targets for the overproduction of the secondary metabolites in this microorganism. Besides including the first metabolic reconstruction of *S. ambofaciens*, the experimental validation of model predictions presented herein underlines the power of *in silico* modeling in the context of metabolic engineering.

## Materials and Methods

### Bacterial Strains, Media and Growth Conditions

*Streptomyces ambofaciens* ATCC 23877 was obtained from the American Type Culture Collection (ATCC). The identity of this strain was verified by sequence analysis using the 16S rRNA genes specific ‘universal’ primers 5′-CAGCAGCCGCGGTAATAC-3′ and 5′-CCGTCAATTCCTTTGAGTTT-3′ (Olsen et al., 1986). The strain was stored in 1 ml cryotubes at -80°C as frozen mycelium in yeast starch (YS) medium containing 15% glycerol at a biomass concentration of approximately 0.25 g dry cell weight (DCW)/ml, or at -20°C as spores in 20% glycerol at a titre of approximately 5 × 10^8^/ml. The composition (per liter) of the media used in this study for *S. ambofaciens* growth and manipulation is here reported. YS broth: 2 g yeast extract, 10 g soluble starch, (18 g agar in YS agar) (pH 7.0); SMII (medium for conjugation experiments): 15 g dextrose, 10 g soybean flour, 0.5 g MgSO_4_ ⋅ 7 H_2_O, 5 g CaCO_3_, 15 g agar; 2XYT (medium for spore resuspension): 16 g tryptone, 10 g yeast extract, 5 g NaCl.

*Escherichia coli* strain DH5α [F^-^ Φ80d *lacZ*ΔM15 *endA1 recA1 hsdR17 supE44 thi-1* λ^-^ *gyrA96* λ (*lacZYA-argF*) *U169*] was used in cloning procedures. This strain was cultivated in Luria-Bertani broth supplemented with 50 μg/ml ampicillin when required. Conjugation experiments were performed with *E. coli* strain GM2929/pUB307::Tn*7*. Lennox Broth (LeB) was used to grow, wash and resuspend this strain. The composition (per liter) of the LeB is as follows: 10 g tryptone, 5 g yeast extract, 5 g NaCl, 1 g glucose.

### DNA Procedures and Construction of Recombinant *S. ambofaciens* Strains

*Streptomyces ambofaciens* total genomic DNA was obtained as described by (Kieser et al., 2000). Manipulation of *E. coli* DNA was performed as indicated by Sambrook and Russell (2001). PCR amplification conditions were 94°C for 5 min followed by 34 cycles of 94°C for 1 min, 65°C for 1 min and 72°C for 1 min, and finally 72°C for 10 min.

Overexpression of *S. ambofaciens ccr* (srm4) (SAM23877_ RS26790 in *S. ambofaciens* ATCC 23877 genome) coding for crotonyl-CoA carboxylase/reductase was achieved by cloning the PCR-amplified coding region into an *ad hoc* engineered expression vector, pNGEM/OriT/P, under the control of *groES* promoter. To construct pNGEM/OriT/P we utilized the *E. coli*–*Streptomyces* shuttle vector pN702GEM3. pN702GEM3 (Fernandez-Abalos et al., 2003) contains the pIJ702 origin of replication (for Streptomycetes), the bifunctional *Neo/Kan* resistance marker from Tn*5* (for both *E. coli* and Streptomycetes), the pUC origin of replication (for *E. coli*) and a polylinker derived from pGEM3Zf(+) (Promega). This vector is replicative but not conjugative in Streptomycetes. To transform it into conjugative vector pNGEM/OriT a DNA fragment containing the *oriT* determinant was amplified by PCR from plasmid pTYM18 (Onaka et al., 2003) using the primer pair OriTHindIII-for and OriTHindIII-rev whose sequence is reported below (*Hind*III restriction sites are underlined):

OriTHindIII-for: 5′-CCGACCAAGCTTCGCCCAACCTGCCATCACGAGATTTC-3′
OriTHindIII-rev: 5′-GAGCTGAAAGCTTCAGAAGCCACTGGAGCACCTC-3′

The amplified fragment was treated with *Hind*III, and the resulting 930 bp DNA fragment was cloned into pN702GEM3 using the *Hind*III restriction site of the polylinker.

To construct the pNGEM/OriT/P plasmid, the *groES* gene promoter (*P_groES_*) region was amplified by PCR using *S. ambofaciens* ATCC 23877 genomic DNA as template, and the primer pair groES-for and groES-rev (*Xba*I and *Nde*I restriction sites are underlined):

groES-for: 5′-GGCCGTTCTAGACCGTCCGGCGTTTCGAGGACGAGG-3′
groES-rev: 5′-GCTGGTTCTAGACATATGCCGACCTCCCCCTTCGGAGATCTCACG-3′

The amplified DNA fragment was digested with *Xba*I, and the resulting 325 bp DNA fragment was inserted into the polylinker of pNGEM/OriT, generating pNGEM/OriT/P.

The crotonyl-CoA carboxylase/reductase (CRR) encoding gene *srm4* (locus tag: SAM23877_ RS26790) was amplified from *S. ambofaciens* ATCC 23877 genomic DNA, using the primer pair CCR-for and CCR-rev (*Nde*I and *Eco*RI restriction sites are underlined):

CCR-for: 5′-GACCGTCATATGCCCGAAAGCCATGCGCAGAGCGCG-3′
CCR-rev: 5′-CCGCACGGAATTCGGAGCACCTGGTGCCGTCACCGGC-3′

The amplified 1475 bp DNA fragment was digested with *Nde*I and *Eco*RI and then ligated to *Nde*I and *Eco*RI –cleaved pNGEM/OriT/P, obtaining the resulting plasmid pNGEM/OriT/P/CCR.

Plasmids pNGEM/OriT/P and pNGEM/OriT/P/CCR were introduced into the *S. ambofaciens* strain by conjugation with *E. coli* GM2929/pUB307::Tn*7*, as described previously Kieser et al. (2000). To allow plasmid selection, conjugation medium SMII was supplemented with kanamycin (25 μg/ml).

### Overproduction and Purification of CCR (Srm4)

To overproduce and purify His6-tagged *S. ambofaciens* CCR (Srm4) in *E. coli*, the entire *ccr* (srm4) gene was amplified by PCR using the pNGEM/OriT/P/CCR construct as template, and using the primer pair CCR-for (above reported) and pETCCR-rev (HindIII restriction site is underlined):

pETCCR-rev: 5′-GAATTCAAGCTTGCGGAACCGGTTGATCGCGTCGA-3′

The amplified DNA fragment was digested with *Nde*I and *Hind*III and then ligated to *Nde*I and *Hind*III –cleaved pET21b, obtaining the resulting plasmid pET/Srm4. pET/Srm4 was then introduced into *E. coli* BL21(DE3) competent cells.

*Escherichia coli* BL21(DE3) harboring pET/Srm4 was grown in 1 l of LB medium supplemented with ampicillin (10 μg ml-1) to A600 = 0.6 at 37°C and 250 r.p.m. Culture was induced by the addition of 0.4 mM IPTG and incubated for 16 h at 20°C and 180 r.p.m. Cells were harvested by centrifugation at 5,500 RCF for 10 min, re-suspended in lysis buffer (20 mM Tris–HCl pH 8.0, 200 mM KCl, 25 mM imidazole, 10% glycerol and 0.1 mg ml^-1^ DNase I). Cells were lysed by passing the suspension through a French Press twice at 1000 psi, and the cell debris were removed by centrifugation at 10,000 × *g* for 20 min at 4°C. The clarified cell lysate was bound to 1 ml of Ni-IMAC resin (Bio-Rad Laboratories) for 1 h at 4°C. The resulting slurry was transferred to a gravity-flow column, and washed twice with 1 ml of lysis buffer. The recombinant protein His6-Srm4 was then eluted with 0.2 ml of elution buffer (20 mM Tris–HCl pH 8.0, 500 mM NaCl, 500 mM imidazole and 10% glycerol), and frozen for storage at -80°C.

### Spiramycin Production Assay

Spiramycin production by *S. ambofaciens* cultures grown in YS broth was assessed by HPLC. At different time intervals, supernatants were filtered through Phenex-RC membrane (0.45 μm; Phenomenex). Four hundred microliter filtrated samples were lyophilized and resuspended in a mixture water-acetonitrile 70:30. The concentration of spiramycins was determined by HPLC (Beckman System Gold Programmable Solvent Module125) equipped with a UV detector (232 nm). The column was a reverse phase LiChrospher^®^RP-8 HPLC Column (Supelco) and the mobile phase was a mixture of 100 mM phosphate buffer (pH 2.3) containing 50 mM sodium perchlorate and acetonitrile 70:30 v/v with a flow rate of 0.8 ml/min. Purified spiramycin (Sigma–Aldrich) freshly dissolved in phosphate buffer (pH 7) was used as standard. Two-tailed paired Student’s *t*-test was used to assess statistical significance of antibiotic titre differences between bacterial strains during the time course. Statistical significance was declared at *p*-value < 0.05.

### Determination of Crotonyl-CoA Carboxylase/Reductase (CCR) Activity

*Streptomyces ambofaciens* cultures (100 ml) were harvested by centrifugation at 10,000 × *g* at 4°C for 20 min. Supernatants were discarded and mycelial pellets were washed twice in homogenization buffer (50 mM potassium phosphate pH 7.5, 1 mM EDTA, 1 mM dithioerythritol (DTE), 10% glycerol, 0.1 mM phenylmethylsulfonyl fluoride). After resuspension in homogenization buffer, mycelial extracts were prepared by mycelial disruption at 2000 psi using a French Press and subsequent centrifugation of the homogenates at 25,000 x *g* for 5 min at 4°C to remove debris. The supernatant was collected and used for the enzyme activity measurement. Protein concentrations were determined by using the colorimetric Bio-Rad colorimetric assays for protein determination with BSA as a standard. Protein extracts were analyzed by sodium dodecyl sulfate polyacrylamide gel electrophoresis (SDS-PAGE) according to standard procedures.

The crotonyl-CoA carboxylase/reductase (CCR) activity was determined as described (Erb et al., 2007) in a reaction mixture containing 50 mM phosphate buffer (pH 7.5), 1 mM EDTA, 1 mM DTE, 1 mM crotonyl-CoA, 30 mM NaHCO_3_ and 0.1–0.3 mg of mycelial extract proteins, or 0.10–0.2 μg of purified CCR protein. Upon equilibration at 30°C for 5 min, 6 mM NADPH was added to initiate the reaction. CCR activity was measured spectrophotometrically by following the decrease in absorbance at 340 nm (𝜀_NADPH_ = 6.22 mM^-1^ cm^-1^), which corresponds to the oxidation of the NADPH. Specifically, the decrease in the absorbance due to CCR-independent NADPH oxidation was excluded from the calculation of CCR activity, by performing an analogous reaction in the absence of crotonyl-CoA. A unit (U) of enzyme activity is the amount of enzyme catalyzing the oxidation of 1 μmol NADPH min^-1^.

### RNA Extraction, Real-Time qPCR and RNAseq Experiments

Total bacterial RNA was extracted from *S. ambofaciens* growing in YS medium at 28°C on a rotary shaker at 200 rpm, by using the RNeasy Mini kit according to the manufacturer’s instructions (QIAGEN). RNase-free DNase I was used to eliminate traces of DNA in the samples, in accordance with the instructions of the manufacturer (Promega, Madison, WI, USA).

Semi-quantitative analyses of the *ccr*-specific transcripts, normalized to 16S rRNA, were carried out by Real-Time qPCR. Total RNAs (1 μg) were reverse-transcribed by using random hexamer (2.5 μM) with Superscript RT (Invitrogen). About 0.1–1% of each RT reaction was used to run real-time PCR on a SmartCycler System (Cepheid) with SYBR^®^Green JumpStart Taq ReadyMix (Sigma–Aldrich) and the oligonucleotide primer pairs 16Suniv-1/16S-univ-2 (specific for 16S rRNA gene) and ccr-rtF/ccr-rtR (specific for *ccr*). The primer sequences (and names) were: 5′-CAGCAGCCGCGGTAATAC-3′ (16Suniv-1); 5′-CCGTCAATTCCTTTGAGTTT-3′ (16Suniv-2); 5′-CGATCGTCACCTGCGCCTCCAC-3′ (ccr-rtF); 5′-CGAAGTGGGTGCCGACGATAC-3′ (ccr-rtR). Primers were synthesized as a service by MWG-Biotech AG Oligo Production. RT-PCR product lengths were 185 bp for 16Suniv-1/16S-univ-2 and 96 bp for ccr-rtF/ccr-rtR. Real-Time qPCR samples were run in triplicate. The Real-Time qPCR conditions were: 30 s at 94°C, 30 s at 60°C, 30 s at 72°C for 35 cycles; detection of PCR products was performed at 83°C. The relative transcript levels were calculated using the 2ΔΔct method (Livak and Schmittgen, 2001).

For RNAseq experiments ribosomal RNAs were depleted starting from 1 μg of total RNA from each of the time points and biological replicates by using the RiboZero Gram positive kit (Epicentre, Illumina), strand specific RNA-seq libraries were prepared by using the ScriptSeq^TM^ v2 RNAseq library preparation kit (Epicentre, Illumina) starting from 50 ng of previously rRNA depleted RNA from each biological replicate and for all the time points analyzed. Then each library was sequenced on a MiSeq Illumina sequencer and 76 bp reads were produced.

### RNAseq Data Analysis

Bowtie 2 (v2.2.6) (Langmead and Salzberg, 2012) was used to align raw reads to *S. ambofaciens* ATCC 23877 genome (GCF_001267885.1). Multi-mapping reads alignment was forced using non-deterministic option to obtain a single mapping locus for the reads located on the terminal arm region (nt 1-202694/8101246-8303940), which is known to be duplicated. High quality reads were selected imposing the following criteria: mapping quality for uniquely mapping reads MAPQ greater than 30 and alignment score greater than -15; for multi-mapping reads the alignment score was set equal or greater than -15. These criteria define high quality reads only those showing a maximum of three variations (mismatches or short indels): the sum of penalty scores assigned to mismatches and short indels should not be greater than -15.

rRNA depletion, strand specificity and gene coverage were evaluated using BEDTools (v2.20.1) (Quinlan and Hall, 2010) and SAMtools (v0.1.19) (Li et al., 2009) to verify the library preparation and sequencing performances (see Supplementary Data Sheet 7 and Table S1). To obtain the raw read counts of protein coding genes on unique chromosomal regions and on the pSAM1 plasmid, strand specific reads overlapping for at least 50% of their length to the genes present in *S. ambofaciens* ATCC 23877 RefSeq annotation were considered in each sample. For protein coding genes located in duplicated regions it was impossible to determine the exact chromosomal origin of the reads. To overcome this problem we reported only one of the two homologs in the final list of genes and the relative raw count is the sum of all the reads aligning to both loci (strand specificity was not considered). The R (R Development Core Team, 2012) package DESeq2 (v1.6.1) (Love et al., 2014) was then used to normalize the counts basing on the median-of-ratio method to estimate the scaling factors.

Time course analysis was performed with maSigPro (Conesa et al., 2006; Nueda et al., 2014). To find the genes whose expression was significantly altered during the time-course, we considered only those expressed in at least five over eight samples. The regression parameters were left to default values (α = 0.05; Q = 0.05) in both the two steps of the analysis, in particular a forward step regression model was adopted in the second step. Significant genes lists were obtained by imposing R-square >0.7, in order to select genes correctly fitting the model as suggested by Conesa et al. (2006). Finally hierarchical clustering was applied to the expression values of the significant genes to individuate 9 time-dependent patterns of gene expression.

The enrichments of functional categories were calculated using Fisher test and BH correction for multiple testing, the categories with an adjusted *p*-value ≤ 0.05 were considered significantly enriched (see next paragraph for functional categories definition).

Raw data are publicly available at Sequence Reads Archive under accession number BioProject PRJNA342588.

### Metabolic Reconstruction and Modeling

The Refseq *S. ambofaciens* ATCC 23877 genome annotation was manually refined by using RAST (Overbeek et al., 2014) and antiSMASH (Weber et al., 2015): functional categories were defined according to RAST classification, and the genes encoding secondary metabolites, annotated by antiSMASH, were manually added to the category of secondary metabolism. This version of the genome annotation is reported in Supplementary Data Sheet 1. A draft metabolic reconstruction of *S. ambofaciens* ATCC 23877 was initially assembled using RAST annotation system with default parameters and then downloaded from the ModelSEED database (Henry et al., 2010). Afterward, this reconstruction was extensively curated by manual inspection following the main steps listed in Thiele and Palsson (2010). Several additional information sources were interrogated, including KEGG (Kanehisa and Goto, 2000), BRENDA (Scheer et al., 2011) and MetaCyc (Caspi et al., 2006). In addition, models of more/less related organisms (*S. coelicolor* and *M. tuberculosis*) were used as reference in a comparative genomics workflow for identifying potentially missing reactions. BLAST (Altschul et al., 1990) searches (adopting the Bidirectional Best Hit criterion) on the *S. ambofaciens* ATCC 23877 genome were carried out to confirm/reject the inclusion of further genes/reactions from related organisms to the *S. ambofaciens* model. The list of *S. ambofaciens* cellular transporters was refined probing the Transporter Classification Data Base (TCDB, Saier et al., 2014). Biosynthetic pathways leading to specific antibiotics known to be produced by *S. ambofaciens* were not initially included in the draft reconstruction. For this reason, extensive bibliographical data mining was carried out to include spiramycin (Richardson et al., 1990; Karray et al., 2007; Nguyen et al., 2010), stambomycin, antimycin, and congocidine into the reconstruction.

Overall, at the end of the manual refinement process, 112 reactions, 80 metabolites and 81 genes were added to the initial reconstruction.

To date, no detailed information on the biomass composition of *S. ambofaciens* ATCC23877 is available in scientific literature. Accordingly, the biomass forming reaction from the *S. coelicolor* A3(2) metabolic model (Borodina et al., 2005) was used to include a reaction accounting for biomass assembly in *S. ambofaciens* ATCC23877 reconstruction.

The FBA method was employed to simulate flux distribution in different conditions. Briefly, FBA is a constraint-based method relying on the representation of the biochemical system under investigation in the form of a stoichiometric matrix S (*m* × *n*), where *m* is the number of metabolites and *n* the number of reactions. FBA is based on the assumption of the cellular pseudo-steady state, according to which the net sum of all the production and consumption rates of each internal metabolite within a cell is considered to be zero. Under this assumption, the system can be described by the set of linear equations:

$$\begin{array}{c} \frac{{\mathrm{dX}}_{i}}{\mathrm{dt}}=\sum_{j=1}^{M}S_{\mathrm{ij}}v_{j}=0,\forall i\in N \end{array}$$

in which X_i_ is the concentration of metabolite i, S_ij_ is the stoichiometric coefficient of the i^th^ metabolite in the j^th^ reaction, v_j_ is the flux of the j^th^ reaction, N the entire set of metabolites and *M* the entire set of reactions.

Upper and lower bounds of flux through each reaction act as further constraints and are expressed as:

$$\begin{array}{c} l_{b}<v_{j}<u_{b} \end{array}$$

where l_b_ and u_b_ are the lower and upper limits for reaction j, respectively. Finally, FBA exploits linear programming to determine a feasible steady state flux vector that optimizes a given objective function (e.g., biomass production).

The reconstructed model was analyzed using COBRAToolbox-2.0 (Hyduke et al., 2011; Schellenberger et al., 2011) in MATLAB^®^R2009b (Mathworks Inc.). Gurobi 5.6^1^ and GLPK 4.32^2^ solvers were used for computational simulations presented herein.

### Pareto Optimality Calculation

We calculated the growth dependencies between biomass and antibiotics biosynthesis using Pareto optimality as described in Heinken et al. (2013). We built Pareto optimum curves by (i) determining the minimal and maximal flux through biomass assembly reaction, (ii) fixing the flux through this reaction at different steps spanning minimal to maximal flux and (iii) maximizing the flux through each antibiotic synthesis reaction for each step. The procedure was repeated with biomass and antibiotics assembly reactions exchanged.

### *In Silico* Identification of Overexpression Targets for Spiramycin Overproduction

The FSEOF algorithm for flux scanning based on enforced objective flux was implemented for the selection of gene targets to be amplified as described in Choi et al. (2010). Briefly, initial fluxes were calculated by constraint-based flux analysis using the objective function of maximizing biomass formation and setting (i) upper and lower boundaries to exchange reactions as to simulate growth in complex medium, (see below) and (ii) the spiramycin production rate to the experimentally measured value of 0.0017 mmol^∗^h^-1^g^-1^. Next, the objective function was set to spiramycin production and the theoretical maximum product formation rate was calculated. Afterward, FSEOF was performed under the objective function of maximizing cell growth while the spiramycin production rate is gradually enforced from the flux value used in the first simulation to the theoretical maximum production rate obtained with the second simulation (i.e., performing ten different model optimizations). Candidate target genes were then collected identifying those fluxes that increased upon the application of the enforced objective flux without changing the reaction’s direction. More specifically, we selected those reactions (and corresponding coding genes) showing an increased flux in at least one of the 10 simulations, without changing the reaction’s direction. Please refer to Choi et al. (2010) for a detailed and formal explanation of the FSEOF algorithm.

## Results and Discussion

### Growth of *S. ambofaciens* ATCC 23877 and Spiramycin Production Kinetics

**FIGURE 1:**
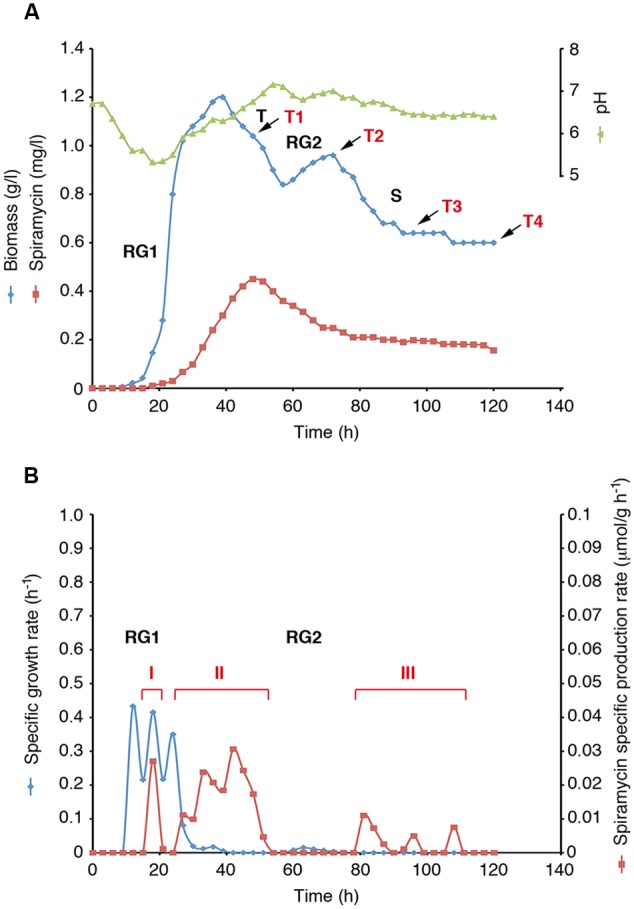
***Streptomyces ambofaciens* ATCC 23877 growth curve and spiramycin production kinetics. (A)** Biomass, pH and spiramycin production curves. Growth phases (RG1, T, RG2 and S) and the four sampling points for RNA-seq analysis is indicated (T1 to T4). **(B)** Growth rate and spiramycin specific productivity. Phases with high specific spiramycin production rates are shown (I, II, III).

Mycelial growth and spiramycin production were measured during *S. ambofaciens* ATCC 23877 growth in shake flask batch cultivation using complex YS medium (**Figure 1** and Supplementary Data Sheet 2). Biomass data displayed the typical two-stage growth curve of actinomycetes consisting of an initial stage of rapid growth (RG1) (12–40 h), a transition phase of stalled growth (T) (40–60 h), a secondary growth phase (RG2) (60–72 h) and a stationary (S) phase (72–120 h) (**Figures 1A,B**). The analysis of growth kinetics also showed that during RG1 specific growth rates (μ_max_) were not constant over time but exhibited three distinct peaks at 12, 18, and 24 h, respectively (**Figure 1B**). Spiramycin production started at 18 h and reached the highest titre in the medium at 48 h (**Figure 1A**). Then its concentration decreased progressively till 78 h and afterward it remained nearly constant till 117 h (**Figure 1A**). The evaluation of specific production rates (*q_p_*) led us to distinguish three distinct phases of spiramycin production (**Figure 1B** and Supplementary Data Sheet 2): a brief pulse of antibiotic production culminating at 18 h (phase I) during RG1, a robust and prolonged phase of production (between 27 and 51 h) (phase II) starting during RG1 and lasting over almost the entire T phase, and a third phase of production (between 81 and 108 h) (phase III) starting after RG2 and lasting over almost the entire S phase. Notably, production phases II and III seem to be preceded by growth rate peaks and are characterized by either growth rate decline (phase II) or no growth (phase III) demonstrating a strict interconnection between growth pulses and activation of spiramycin production (Supplementary Data Sheet 2).

### Time-Resolved Transcriptomics of *S. ambofaciens* ATCC 23877

To gain a better insight into the physiological and genetic mechanisms governing mycelial growth and antibiotic production, after RG1, global transcriptional analysis was carried out by RNA-Seq technology. For RNAseq experiments, samples were collected from shake flask batch cultures in YS medium at four different time points (T1 = 48 h, T2 = 72 h, T3 = 96 h, and T4 = 120 h) for RNA extraction. Two biological replicates were analyzed for each time point.

The maSigPro bioconductor package (Conesa et al., 2006) was adopted to analyze the RNA-Seq time series dataset starting from the normalized gene counts obtained after DESeq2 (Love et al., 2014) standard normalization (see Material and Methods). maSigPro was initially developed for the analysis of single and multi-series time course microarray experiments, and was recently adapted to deal also with Next Generation-Sequencing (NGS) series of data (Nueda et al., 2014). This package follows a two-step regression strategy to find genes with significant temporal expression changes and/or significant differences between experimental groups. Following the first step of regression the software individuated 3551 significant genes in our dataset and then, in a second step, it refined this number to a total of 1186 variable genes, 598 upregulated and 588 downregulated during the time course. Among them we found 30 upregulated genes coding for biosynthetic enzymes for secondary metabolites, most of which involved in butyrolactone, stambomycin, and alpomycin/kinamycin production; while among the downregulated genes 37 participate in spiramycin production. Moreover 34 genes were positioned on the duplicated arm region where the cluster of genes coding for alpomycin and kinamycin production is located, while 32 genes were expressed by the extrachromosomal plasmid pSAM1 (Supplementary Data Sheet 3).

**FIGURE 2:**
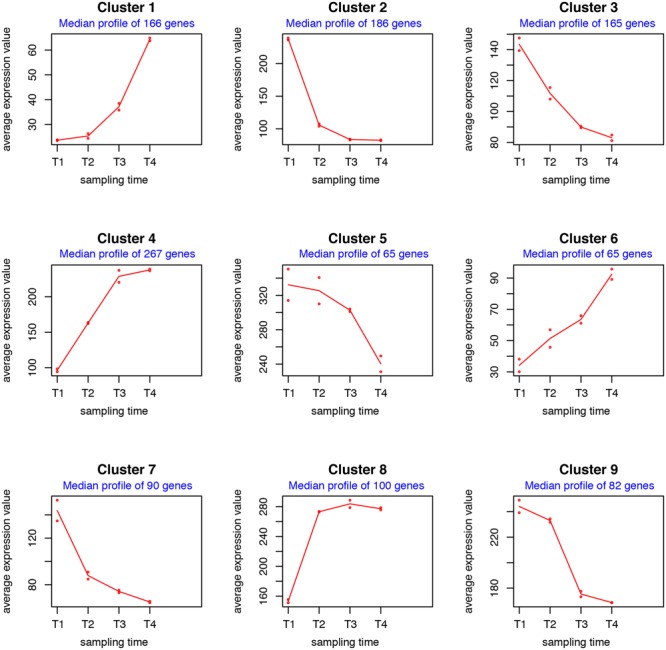
**maSigPro analysis of RNA-Seq time series dataset.** The median profile of each cluster was inferred from the expression patterns shown in Supplementary Data Sheet 7 and Figure S1.

The 1186 variable genes were finally subdivided into nine clusters according to their gene expression profiles (Supplementary Data Sheets 3, 7 and Figure S1) and median profile was then inferred for each cluster (**Figure 2**). Clusters **1, 4, 6**, and **8** consist of genes whose expression levels increase during the time course, although with distinct patterns and basal expression levels. In particular the expression levels of genes in cluster **8** has high basal expression at T1 (48 h), rapidly increased from T1 (48 h) to T2 (72 h) and then remained roughly constant throughout T3 (96 h) and T4 (120 h). Expression levels of genes in cluster **4** also exhibited high basal expression at T1 (48 h), progressively increased from T1 to T3 and remained high at T4. These two clusters are significantly enriched in functional categories “cell division and cell cycle” (cluster **4**), “cell wall and capsule” (clusters **4** and **8**), “DNA metabolism” (clusters **4** and **8**), and “sulfur metabolism” (cluster **8**) (Supplementary Data Sheet 3). The enrichment in these categories is consistent with growth kinetics indicating a secondary growth phase (RG2) between 60 and 72 h (**Figure 1**).

Genes in clusters **1** and **6** showed low expression levels at T1 (48 h) and gradually reached their highest expression level during T4, although with different kinetics. Cluster **1** is enriched in the functional category “secondary metabolism” (Supplementary Data Sheet 3). In particular, it contains five type I polyketide synthase (PKS)-encoding genes involved in stambomycin biosynthesis, and several regulatory genes (belonging to TetR and SARP families) of the alpomycin/kinamycin gene cluster. Genes involved in butyrolactone biosynthesis also belong to this cluster (Supplementary Data Sheet 3). Cluster **6** is enriched in the functional category “fatty acid, lipid, isoprenoids,” but it also contains four type I PKS-encoding genes of the stambomycin gene cluster (Supplementary Data Sheet 3). This result indicates that all nine PKSs-encoding genes involved in the biosynthesis of the large (51-membered) glycosylated macrolide stambomycin (Laureti et al., 2011) achieve the highest expression levels in the late stationary phase (S). This finding is of interest for management of spiramycin production. In fact, the biosynthetic pathways leading to spiramycin and stambomycin compete with each other for common metabolic precursors (i.e., malonyl-CoA and methylmalonyl-CoA) during the assembly of the macrolide scaffold.

It is also noteworthy that the up-regulated clusters **1, 4, 6**, and **8** contain more than 95% of the genes modulated on pSAM1 indicating a global gene expression increase of this extrachromosomal element during late growth.

Such a competition is possibly alleviated by differential temporal expression patterns of the genes involved in spiramycin and stambomycin biosynthesis (Supplementary Data Sheet 3) as suggested by maSigPro analysis that assigned 4 spiramycin biosynthetic genes to cluster **2**, 16 genes to cluster **3**, 5 genes to cluster **7** and 2 genes to cluster **9**. Indeed, although with different kinetics, the spiramycin genes belonging to the clusters **2, 3, 5, 7**, and **9** exhibited a general trend of down-regulation during the time course while the stambomycin genes increased their expression level during the time course (**Figure 2** and Supplementary Data Sheet 7 and Figure S1). Consistently with growth kinetics (**Figure 1**), clusters **2** and **3** contains many genes coding for ribosomal proteins and translation factors, and genes involved in central carbon (and energy) metabolism. Indeed, all these genes are expected to be strongly down-regulated beyond the RG1. Cluster **2** is enriched in the functional categories “membrane transport,” “virulence, disease and defense,” while cluster **3** is enriched in genes involved in “secondary metabolism” (essentially, spiramycin biosynthesis). Cluster **7** exhibits an enrichment in the category “fatty acid, lipid, isoprenoids,” while clusters **5** and **9** exhibit no significant enrichment in any considered functional categories.

### The Metabolic Reconstruction of *S. ambofaciens* Is in Agreement With Experimental Growth Data on Defined Media

A *S. ambofaciens* ATCC 23877 draft reconstruction was obtained from ModelSeed as described in “Material and Methods.” As it is usually expected for automatically reconstructed metabolic networks, this draft model failed in predicting *S. ambofaciens* growth, both on known defined media and on an arbitrarily complex one. Thus, the model underwent a manual refinement process which led to the inclusion of 112 reactions, 80 metabolites and 81 genes, the re-formulation of the biomass assembly reaction (following the one available for *S. coelicolor*) and the inclusion of gap-filling reactions (i.e., not gene-encoded) to reconcile *S. ambofaciens* growth on defined minimum media (see below). The obtained reconstruction was named iMF1244 according to the standard nomenclature and its main features are reported in **Table 1**. The SBML-formatted version of iMF1244 is available as Supplementary Data Sheet 4.

**Table 1:** Main features of the *Streptomyces ambofaciens* ATCC23877 metabolic reconstruction.

*S. ambofaciens* ATCC23877 genome	
Genome size (bp)	8099129
N. of protein encoding genes	7208
***iMF1244* model**	
N. of genes (% of coding genes)	1244 (17%)
N. of reactions	1473
Gene-associated	1210
Non gene-associated (Exchange reactions)	263 (144)
N. of metabolites	1283

**FIGURE 3:**
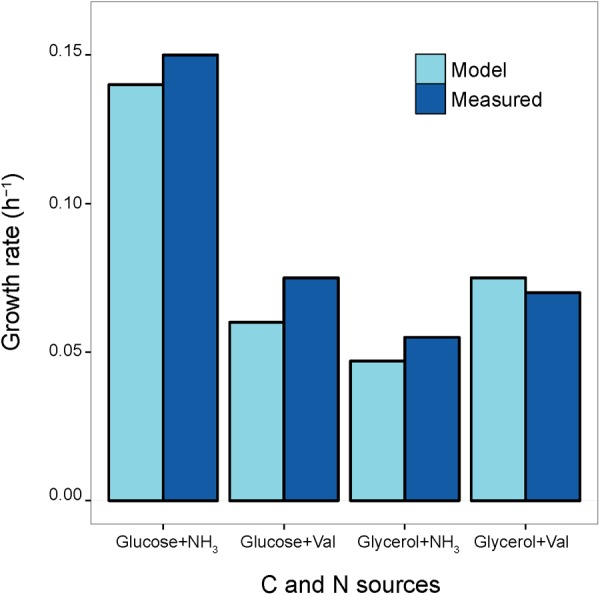
**Growth Rates.** Comparison between model-predicted growth rates and experimentally determined ones on four minimum growth media providing different Carbon (C) and Nitrogen (N) sources (glucose and ammonium, glucose and valine, glycerol and ammonium, glycerol, and valine). The values reported by the blue bars correspond to measurements obtained from previously published works (Lounès et al., 1995, Lounès et al., 1996a,b; Colombie et al., 2005)

The reliability of the reconstructed *S. ambofaciens* model was inferred by comparing the predicted growth rates on a set of different minimal media against a set of experimentally determined ones available from previous studies (Lounès et al., 1995, 1996a,b; Colombie et al., 2005). Accordingly, *in silico* minimal growth media were defined using exchange reactions present in the model, and biomass optimization was selected as the model objective function (O.F.). More in detail, lower bounds of exchange reactions accounting for all the salts present in the experimental medium were set to -10 mmol g^-1^ × h^-1^, in order to mimic non-limiting conditions. The carbon source of each available minimum growth medium was then chosen as the unique carbon source of this *in silico* medium and its uptake flux was set to the corresponding experimentally determined value. The compositions of all the simulated *in silico* media are reported in Supplementary Data Sheet 7 and Table S2. The growth rates that were inferred by the *in silico* simulation were then compared to those experimentally determined, revealing an overall good agreement (Pearson’s product-moment correlation 0.97, *p*-value 0.02, **Figure 3**). This indicates that the model should be able to reproduce a realistic picture of the *S. ambofaciens* ATCC 23877 metabolic landscape.

Afterward, we tested the response of the model in a simulated complex medium. The assimilation of yeast extract (YE) amino acids and its effect on the production of spiramycin in culture of *S. ambofaciens* has been extensively analyzed by Benslimane et al. (1995). In this work the authors showed that in a YE medium a specific growth rate (μ_max_) of 0.15 h^-1^ was soon established after setting up the culture. The specific growth rate then decreased sharply to reach zero value at 24 h and rose up again when a second growth phase with a μ_max_ of 0.012 h^-1^ was observed. During this second growth phase, spiramycin biosynthesis was observed, with a specific production rate (*q*_p_) of 1.46 mg^-1^h^-1^g^-1^ (corresponding to 0.0017 mmol^∗^h^-1^g^-1^). The growth on YE of *S. ambofaciens* was characterized by the consumption of all the amino acids available in the medium (Benslimane et al., 1995). Accordingly, to account for such behavior, we added to the model all the 20 amino acids exchange and transport reactions. Since no detailed information is available concerning the single amino acids uptake rates in the second growth phase on YE, each uptake flux was arbitrary set to the value of 0.015 mmol^∗^h^-1^g^-1^. In such conditions, setting the growth rate exactly to the measured value of 0.012 h^-1^, the model predicts a spiramycin production rate of 0.0016 mmol^∗^h^-1^g^-1^, which resembles the one experimentally determined (0.0017 mmol^∗^h^-1^g^-1^).

In a typical scenario, the metabolic pathways for the production of secondary products (such as antibiotics) should compete for the pool of available metabolic intermediates with all the other cellular biosynthetic processes (e.g., biomass formation and/or homeostasis maintenance). This is typically accounted for by the so-called Pareto front between the two objectives (Marler and Arora, 2004). Pareto front refers to the set of resources allocation that is Pareto efficient, i.e., the situation in which it is impossible to make any one objective better off without making at least one individually worse. In other words, a pair of objective functions (such as a biomass and antibiotic assembly reactions) can be balanced to find the set of optimal solutions for which one objective can only be improved at the expense of the other. Recently, it has been shown that cells’ metabolism operates close to the Pareto-optimal (Schuetz et al., 2012) and that the calculation of Pareto fronts represents a technique that is more effective than weighting the objectives (Gevorgyan et al., 2011), especially when genetic algorithms are used to approximate the front (Boghigian et al., 2010; Angione and Lió, 2015).

Accordingly, we computed (see Materials and Methods for details) Pareto optimality for each of the four antibiotics that were included in the reconstruction. As shown in Supplementary Data Sheet 7 and Figure S2 the maximum theoretical yields were observed in the condition of μ = 0 and were 0.0052, 0.0105, 0.00012 and 0.00086 mmol^∗^h^-1^g^-1^ for spiramycin, antimycin, stambomycin and congocidine, respectively. Afterward, the model (correctly) predicts a decrease in the production rates of each antibiotic as a consequence of an increase of the growth rate. These results, together with the consistence of growth rates prediction, support the iMF1244 as being a reliable reconstruction of the metabolism of this *S. ambofaciens* ATCC 23877.

### Expression Data Integration With Metabolic Modeling Identifies System-Level Metabolic Trends

**FIGURE 4:**
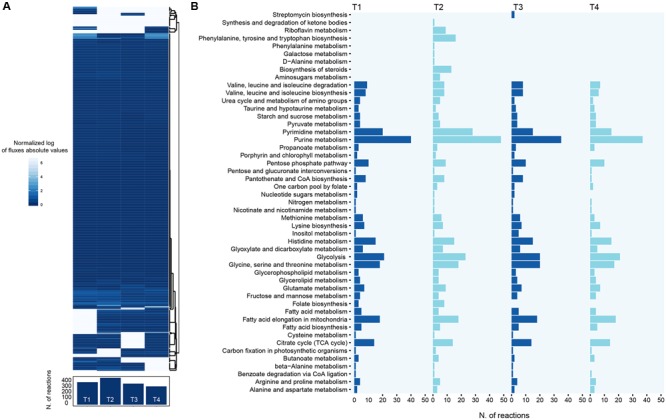
**Distribution of metabolic fluxes. (A)** Heatmap showing the predicted flux carried by each reaction in the model across each of the time points. Also, the number of flux-carrying reactions for each point is shown. **(B)** The number of active reactions (i.e., reactions predicted to carry flux) is reported for the major pathways in the reconstruction, for each of the four time points analyzed.

Here we used our reconstruction to provide a global description of *S. ambofaciens* metabolism in the analyzed time points, by integrating gene expression data with constraint-based metabolic modeling. In general, many methods to achieve this task have been developed to date (Machado and Herrgard, 2014). However, a recent work (Kim et al., 2016) showed that, among them, iMAT (Shlomi et al., 2008) led to more accurate predictions in the analysis of *Streptomyces coelicolor* metabolic model. Briefly, iMAT uses gene expression values to divide reactions into two groups: highly and lowly expressed. The algorithm next seeks to find the flux distribution that maximizes the consistency with this classification. Accordingly, we first computed the first and third quartiles for RPKM values in each time point to define down- and up-regulated genes, respectively (obtained values are reported in Supplementary Data Sheet 7 and Table S3). Next, we performed four different FBA optimizations (one for each of time points analyzed) using iMAT to constrain fluxes across reactions according to gene expression values. For each optimization the flux through biomass assembly and spiramycin production reactions was set to the values experimentally determined (see **Figure 1** and Supplementary Data Sheet 7 and Table S4) and the resulting fluxes distribution analyzed. To account for the cellular maintenance cost, an arbitrary cellular growth rate of 1e^-10^ h^-1^ was set in those cases in which no growth had been experimentally observed. Results obtained, however, were not affected when this arbitrary cellular growth rate was replaced by a simulated ATP maintenance cost. In this case a distribution of overlapping fluxes was observed. **Figure 4A** shows the flux across each reaction for every time step and the number of flux-carrying reactions.

Our model predicts that, on average, 392 reactions are active across all the time points (standard deviation, *SD* = 63.4). Accordingly, around 1000 reactions are predicted to be inactive in each time point (73% of the model reactions, on average). This is in agreement with the fact that the bacterium is growing in a complex medium and thus can find in the surrounding environment most of its cellular building blocks. Interestingly, the second time point is the one displaying the highest number of flux-carrying reactions (475), in agreement with the fact that, unlike the other time points, *S. ambofaciens* is actively producing biomass in this moment of the growth phase. After this time point, *S. ambofaciens* experiences a decreasing trend in the number of flux-carrying reactions (**Figure 4**) reflecting with both the reduced growth rate and lower spiramycin productivity observed *in vivo* in last phases of the growth.

From a functional viewpoint, our modeling predicts that the set of active pathways is largely shared by the four time points (**Figure 4B**). Indeed, a similar scheme of reactions is apparently carrying flux across all the time points, including the main central routes of *S. ambofaciens* metabolism (e.g., purine and pyrimidine metabolism, glycolysis, TCA cycle). This suggests the absence of a massive metabolic reprogramming in the four time points analyzed, i.e., the final part of *S. ambofaciens* growth. The most evident exception to this general trend is represented by the second time point, the one also showing the highest number of active reactions. Our functional annotation of reactions predicted to be carrying flux suggests that this difference is due to the activation of four main pathways i.e., amino-sugar metabolism, steroid biosynthesis, phenylalanine, tyrosine and tryptophan biosynthesis, riboflavin metabolism (**Figure 4B**). Thus, according to our simulations, these pathways (or part thereof) play a role when the cell is trying to achieve two objective functions simultaneously, i.e., biomass and antibiotic synthesis. The activation of riboflavin metabolism and a consequent increase in FAD cofactor availability, for example, is consistent with an increased cellular demand of acetyl-CoA (a substrate for the synthesis of many spiramycin intermediates) that, in turn, can be obtained through the FAD-dependent degradation of fatty acids. Similarly, the use of amino sugar metabolism might be propaedeutic to the recycling of nucleotide hexoses (e.g., glucose-1P), valuable precursors for the sugar moieties of the spiramycin molecule.

All these data show how changes in gene expression may redirect the cellular metabolic fluxes within the cell, leading to activate specific routes that, in turn, may represent key nodes in the utilization of intermediates that are common to multiple cellular objectives (biomass formation and spiramycin synthesis in this case). The hypothetical scenario depicted here indicates both FAD production and nucleotide sugars recycling as key factors in allowing the cell to achieve the imposed double optimization.

### Metabolic Modeling Identifies Potential Overexpression Targets for Spiramycin Overproduction

We next exploited the iMF1244 metabolic reconstruction for the genome-wide identification of gene amplification targets potentially allowing spiramycin overproduction in *S. ambofaciens.* To this purpose we used the FSEOF approach described in Choi et al. (2010), as reported in Section “Materials and Methods.” From the set of identified potential targets, we excluded from further consideration those reactions that (i) were added to the model for gap-filling, (ii) were involved in the import (or export) of nutrients into the system for modeling purposes (i.e., exchange reactions) and (iii) were either encoded by multiple genes (enzymatic complexes) or, at least, two alternative genes (paralogs) in the genome. In all these cases, no sufficiently detailed clues on the gene(s) to be amplified for spiramycin overproduction could be derived. Following these selection criteria, our approach led to the identification of 65 potential reactions (corresponding to 54 genes) whose increased flux may lead to an increased flow through spiramycin synthesis pathway. The complete list of the reactions identified is reported in Supplementary Data Sheet 5.

A large fraction of this set of reactions is involved in the synthesis of precursors of the macrolactone backbone of spiramycin. These include, for example, glycerone phosphate (produced by sn-glycerol-3-phosphate: NAD+ 2-oxidoreductase) involved in the biosynthesis of methoxymalonyl-ACP, crotonoyl-CoA (the product of butanoyl-CoA: oxygen 2-oxidoreductase) precursor of ethylmalonyl-CoA and acetyl-CoA, the substrate of 3-oxopropanoate: NAD+ oxidoreductase for the generation of malonyl-CoA. Reactions leading to the production of intermediates necessary for the synthesis of the deoxyhexose sugars embedded in the spiramycin molecule (e.g., alpha-D-glucose 1-phosphate 1,6-phosphomutase responsible for the conversion of D-glucose 1-phosphate into D-glucose 6-phosphate) were also identified.

Among the proposed targets, we selected the reaction carried out by crotonyl-CoA carboxylase/reductase and presumably involved in the synthesis of ethylmalonyl-CoA. The spiramycin macrolactone backbone is synthesized by a PKS that, apart from the most common polyketide precursors, malonyl-CoA and methylmalonyl-CoA, was predicted to incorporate the less common precursor, ethylmalonyl-CoA, and the functionalized extender unit, methoxymalonyl-CoA (Kuhstoss et al., 1996; Karray et al., 2007). In particular, the ethylmalonyl-CoA is used as building block to assemble only a few known polyketides, including the polyether antibiotic monensin A, whose biosynthetic pathway was the first established pathway to require ethylmalonyl-CoA (Liu and Reynolds, 1999).

Although there is more than one pathway to ethylmalonyl-CoA, the pathway involving the crotonyl-CoA carboxylase/reductase (CCR) (Erb et al., 2007) (commonly referred to as “the ethylmalonyl-CoA pathway”) appears to be the dominant source of this precursor for polyketide biosynthesis (Wilson and Moore, 2012). This pathway was originally described in purple non-sulfur bacterium *Rhodobacter sphaeroides* and then discovered also in methylotrophic bacteria such as *Methylobacterium extorquens* and in *Streptomyces* spp. In the ethylmalonyl-CoA pathway a C4-compound, acetoacetyl-CoA, derived from two molecules of acetyl-CoA, is converted to the C5-compound mesaconyl-CoA (2-methylfumaryl-CoA) that is then transformed to (*2R,3S*)-β-methylmalyl-CoA by hydration and is finally cleaved to glyoxylate and propionyl-CoA. The key enzyme of the pathway is CCR, which simultaneously carboxylates and reduces the C4-compound crotonyl-CoA, forming the C5-compound (*2S*)-ethylmalonyl-CoA (herein indicated as “ethylmalonyl-CoA”). As illustrated in Supplementary Data Sheet 7 and Figure S3, the ethylmalonyl-CoA and its direct metabolic precursor crotonyl-CoA lies at the crossroad between key metabolic pathways devoted to energy metabolism and energy storage, lipid and amino acid metabolism and secondary metabolism.

In *S. ambofaciens* the ethylmalonyl-CoA is used as a building block to synthesize the polyketide backbone of spiramycin and antimycin, and the full set of genes necessary for a functioning ethylmalonyl-CoA pathway is clustered together in the *S. ambofaciens* chromosome not very distant from the spiramycin biosynthetic cluster. The cluster comprises the ORFs: SAM23877_RS28800 coding for methylsuccinyl-CoA dehydrogenase (*msd* gene); SAM23877_RS28805 coding for mesaconyl-CoA hydratase (*mcd* gene); SAM23877_RS28810 coding for L-malyl-CoA/beta-methylmalyl-CoA lyase (*mclA* gene); SAM23877_RS28815 encoding ethylmalonyl-CoA mutase (*ecm* gene); SAM23877_RS28820 encoding the CCR (*ccr* gene); SAM23877_RS28825 coding for a TetR family transcriptional regulator; SAM23877_RS28830 coding for 3-hydroxybutyryl-CoA dehydrogenase (Supplementary Data Sheet 7 and Figure S3).

As well as in other polyketides gene clusters, a secondary copy of the CCR-encoding gene is located in both spiramycin (SAM23877_RS26790, *srm4*) and antimycin (SAM23877_RS01780) biosynthetic gene clusters. RNAseq data showed that under tested experimental conditions all three CCR-encoding genes were poorly expressed, the gene RS26790 has an expression level higher than the gene RS28820 which is more expressed than the gene RS01780 (RS26790 > RS28820 > RS01780), among them only the gene SAM23877_RS26790 (*srm4*) shows an expression level above the baseline threshold (RPKM > 10) in all the time points analyzed, thus allowing us to consider it effectively expressed along the growth curve (**Table 2**). Although the CCR enzymatic activity of the *ccr* paralog gene products has not been proven so far, their role is likely to synchronize ethylmalonyl-CoA synthesis from acetoacetyl-CoA with polyketide assembly, and/or to increase intracellular ethylmalonyl-CoA levels in order to ensure adequate levels of the precursor during polyketide biosynthesis. Therefore, the carbon flow through the ethylmalonyl-CoA pathway was consistently associated with spiramycin productivity by our metabolic model.

**Table 2:** Expression of CCR-encoding genes as deduced by RPKM values from RNAseq analysis.

Timepoint	Time (hours)	SAM23877_ RS26790 (RPKM value)	SAM23877_ RS28820 (RPKM value)	SAM23877_ RS01780 (RPKM value)
T1	48	13.5	8.5	3.5
T2	72	16	7	6
T3	96	16	10.5	6.5
T4	120	32	26.5	18.5

### Manipulation of the Ethylmalonyl-CoA Metabolic Node Leads to Spiramycin Overproduction

**FIGURE 5:**
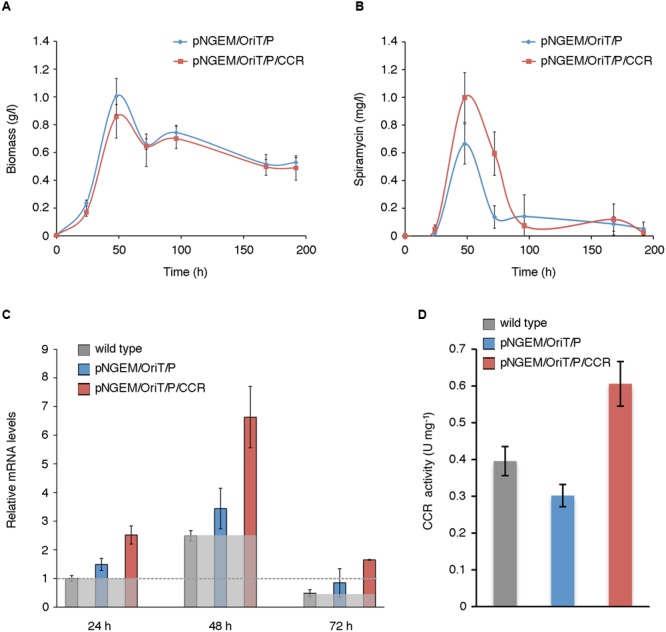
**Genetic manipulation of the ethylmalonyl-CoA metabolic node. (A,B)** Mycelial growth **(A)**, and spiramycin production **(B)** of wild type and trans-conjugant strains harboring pNGEM/OriT/P/CCR or control plasmid pNGEM/OriT/P grown in YS medium. Values represent mean ± standard deviation (SD) (bars) from seven independent experiments (biological replicates). In **(B)**, each assay was carried out with triplicate samples (technical replicates). The variability associated with the technical replicates (including extraction and analytic procedure with samples processed the same day) was less than 0.3%. **(C,D)** *ccr* (*srm4*) mRNA levels **(C)** and CCR enzymatic activity **(D)** in wild type and recombinant strains. In **(C)**, the expression levels of *ccr* (*srm4*) (means from three independent experiments), compared with those of the wild type strain at 24 h arbitrarily assumed equal to 1, are reported. Bars represent standard deviations. The sheer gray boxes delineate the expression levels of *ccr* (*srm4*) in the wild type strain for each time point; the gray dotted line marks the expression levels of *ccr* (*srm4*) of the wild type strain at 24 h taken as calibrator for the histogram representation. In **(D)** the CCR enzymatic activity was determined in crude mycelial extracts of wild type and *trans*-conjugant strains harboring pNGEM/OriT/P/CCR or control plasmid pNGEM/OriT/P grown in YS medium for 48 h. Values represent means from three independent experiments. Bars represent standard deviations.

To validate this prediction, we planned to over-express the *ccr* paralog *srm4* of the spiramycin gene cluster in *S. ambofaciens.* However, we first verified that *srm4* actually codes for an enzyme with CCR activity. To this purpose, the His6-tagged *S. ambofaciens* CCR (Srm4) protein was overproduced in *E. coli* BL21(DE3) strain and purified (Supplementary Data Sheet 7 and Figure S4). Then its CCR enzymatic activity was determined. The results demonstrated an enzymatic activity of 449 ± 11 U mg-1. To over-express *ccr* (*srm4*), the gene was placed under the control of the *PgroES* promoter (Luo et al., 2015) in pN702GEM3, a multicopy *E. coli*-*Streptomyces* shuttle plasmid vector (Supplementary Data Sheet 7 and Figure S5). The pNGEM/OriT/P/CCR or the control plasmid pNGEM/OriT/P were then introduced into *S. ambofaciens* ATCC 23877 by intergeneric conjugation with *E. coli*, and mycelial growth (**Figure 5A**), spiramycin production (**Figure 5B**) *ccr* (*srm4*) mRNA levels (**Figure 5C**), and CCR enzymatic activity in crude cell extracts (**Figure 5D**) were analyzed in the trans-conjugant strains grown in YS medium. With respect to growth kinetics, compared to control strain (harboring pNGEM/OriT/P) the *ccr*-over-expressing strain exhibited major differences during the RG1 with about 14.6% lower final biomass values but similar growth rates (0.0697 vs. 0.0622 between 24 and 48 h) (**Figure 5A** and Supplementary Data Sheet 6b).

Strain manipulation did not alter significantly either soluble protein content (expressed as mg protein/g DCW) or electrophoretic pattern as determined, respectively, by Bio-Rad colorimetric assays and SDS-PAGE (Supplementary Data Sheet 7 and Figure S6). In each time point the *ccr* (*srm4*) mRNA expression levels of the strain over-expressing *ccr* (pNGEM/OriT/P/CCR), were compared at the same time both with the expression levels of the wild type and of the strain with control plasmid (pNGEM/OriT/P). As shown in **Figure 5C** the strain over-expressing *ccr* (*srm4*) exhibited an average maximum increase in the mRNA levels of 2–3-fold when compared respectively with wild type or with the control plasmid strain, thus indicating a moderate gene overexpression at each time point (**Figure 5C**). It is noteworthy that during the time course, in all the three strains *ccr* (*srm4*) mRNA levels reached the highest values of expression at 48 h. So in the light of these results we can infer that the percentage of the overexpressed Srm4 protein in the total soluble *S. ambofaciens* proteome is therefore almost negligible.

In the *ccr*-over-expressing strain spiramycin productivity was enhanced during the RG1 and T phases. Compared to control strain spiramycin specific production rates (*q_p_*) were increased by a factor of 3.63 at 24 h (due to early triggering of antibiotic biosynthesis) and 1.52 at 48 h (Supplementary Data Sheet 6b), but antibiotic titres were only modestly increased by a factor of 1.503 at 48 h (i.e., an average increase of 50.3% with a standard deviation of 36.5%, *p*-value = 0.01078), when spiramycin titres reached the highest values during the time course. Antibiotic titres were instead significantly increased by a factor of 4.336 at 72 h (i.e., an average increase of 333.6% with a standard deviation of 235.6%, *p*-value = 0.00024) (**Figure 5B** and Supplementary Data Sheet 6a). In contrast, no statistically significant differences were observed during the late phase of antibiotic production (during RG2 and S phases).

The achieved increase was rather limited when compared to that obtained with similar metabolic engineering strategy that resulted in about 2- and 1.8-fold increase in actinorhodin production by *S. coelicolor*A3(2) strains overexpressing, respectively, the ribulose 5-phosphate 3-epimerase and the NADP-dependent malic enzyme (Kim et al., 2016). However, the increase was comparable to those obtained in other streptomycetes by genetic modification of single targets (Huang et al., 2012, 2013). For instance, in *Streptomyces tsukubaensis* 35, 40, or 50% improvement in FK506 (a 23-membered polyketide macrolide also known as tacrolimus) production could be achieved, respectively, by overexpression of *zwf2* (coding for glucose-6-phosphate dehydrogenase), *accA2* (coding for acetyl-CoA carboxylase) or *dahp* (coding for 3-deoxy-D-arabino-heptulosonate-7-phosphate synthase). However, the combined effect of *gdhA*-deletion and *dahp*-, *accA2*-, *zwf2*-overexpression enhanced the concentration of FK506 up to 1.47-fold in fed-batch fermentations (Huang et al., 2013). In another streptomycete, such as for example *Streptomyces roseosporus*, co-overexpression of *zwf2, dptI*, and *dptJ* genes resulted in a 34.4% higher daptomycin concentration compared with the parental strain (Huang et al., 2012). These comparisons indicate that the achieved increase may not be irrelevant, and that spiramycin production may be further enhanced by the combined effect of *ccr*-overexpression, and genetic manipulation of other identified targets and/or improvement of fermentation conditions. Preliminary data indicate very long-chain acyl-CoA dehydrogenases, and several enzymes involved in glyoxylate cycle and glycine, serine and threonine metabolism as further good targets for strain improvement.

The results of biochemical assay with crude mycelial extracts demonstrated that the increase in spiramycin productivity paralleled with an increase in CCR activity (**Figure 5D**). In particular, at 48 h of growth, the enzyme activity in the *ccr*-over-expressing strain was about two-fold higher than in control strain (harboring pNGEM/OriT/P). It should be noted also that the order of magnitude of CCR activity that was determined in the protein extracts from *S. ambofaciens* is similar to those assessed in protein extracts from *Rhodobacter sphaeroides* (0.7 U mg^-1^), *Methylobacterium extorquens* (0.8 U mg^-1^) and *Streptomyces coelicolor* A3(2) (0.4 U mg^-1^) (Erb et al., 2007). Altogether these results validate the metabolic model providing evidence that spiramycin production may be increased by genetic manipulation of the ethylmalonyl-CoA metabolic node.

## Conclusion

**FIGURE 6:**
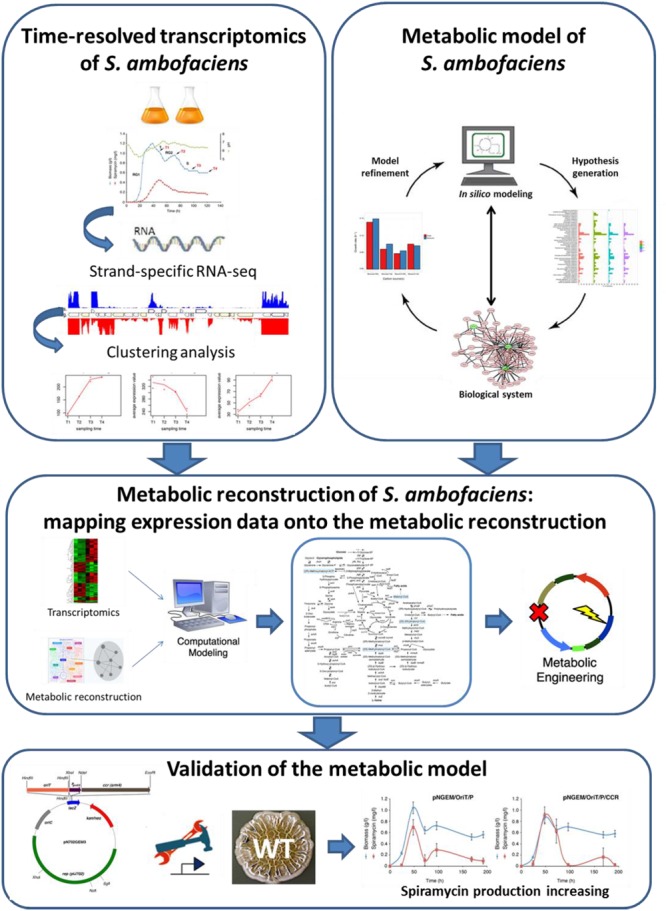
**Schematic representation of the integrated approach Workflow herein proposed**.

In this study we have applied an integrated approach to explore the metabolic landscape of *Streptomyces ambofaciens* with the aim to provide a system-level understanding of its metabolic features, and to identify a list of potential metabolic engineering targets for the overproduction of the secondary metabolites in this microorganism (**Figure 6**).

Our work culminated in the construction of a metabolic model that enabled us to identify key molecular targets for strain improvement. In particular, we proposed a set of genes/reactions that might be preferential targets for metabolic engineering experiments aimed at the overproduction of spiramycin. Notably, we selected one of these targets and we validated the model predictions by providing evidence that spiramycin productivity may be considerably increased by enhancing the carbon flow through the recently described ethylmalonyl-CoA metabolic pathway. This goal was achieved by over-expressing the *ccr* paralog *srm4* in an *ad hoc* engineered plasmid. In the future this strategy will be valuable in reconstructing the genetic and metabolic profiles of other streptomycetes, and of great benefit to the development of industrial overproducers by genetic engineering.

## Author Contributions

PA, GDB, MF, and CP conceived the experimental design; CC, CP, and EP produced RNA-seq data and performed the relative analysis; MF and BM constructed the metabolic model; MF performed all the simulations with the model; PA, FD, DF, GEDB, AT, and MT performed the experimental validation and provided the material for RNA-seq library preparation. PA, MF, CP, EP, and AT wrote the draft of the work. All the authors critically revised and finally approved the manuscript.

## Conflict of Interest Statement

The authors declare that the research was conducted in the absence of any commercial or financial relationships that could be construed as a potential conflict of interest.

## Footnotes

1 http://www.gurobi.com

2 http://www.gnu.org/software/glpk/
