# Supplementary material for: Time-Resolved Transcriptomics and Constraint-Based Modeling Identify System-Level Metabolic Features and Overexpression Targets to Increase Spiramycin Production in Streptomyces ambofaciens
Source: Front Microbiol. 2017 May 12;8:835. doi: 10.3389/fmicb.2017.00835 (PMC5427115; doi:10.3389/fmicb.2017.00835)
Supplement: Supplementary file 7 [file Data_Sheet_7.docx]

***Supplementary Material***

**Time-resolved Transcriptomics and Constraint-based Modelling Identify System-level Metabolic Features and Overexpression Targets to Increase Spiramycin Production in *Streptomyces ambofaciens*.**

**Marco Fondi ^a,1^, Eva Pinatel ^b,1^, Adelfia Talà ^c,1^, Fabrizio Damiano ^c^, Clarissa Consolandi ^b^, Benedetta Mattorre ^a^, Daniela Fico ^d^, Mariangela Testini ^c^, Giuseppe Egidio De Benedetto ^d^, Luisa Siculella ^c^, Gianluca De Bellis ^b^, Pietro Alifano ^c,^* and Clelia Peano ^b,^***

^a^ *Department of Biology , University of Florence, Florence, Italy*

^b^ *Institute of Biomedical Technologies, National Research Council, Segrate, Milan, Italy*

^c^ *Department of Biological and Environmental Sciences and Technologies, University of Salento, Lecce, Italy*

*^d^ Laboratory of Analytical and Isotopic Mass Spectrometry, Department of Cultural Heritage, University of Salento, Lecce, Italy*

^1^ These authors contributed equally to this publication and should be considered co-first authors.

* Corresponding authors

**Correspondence:**

Dr. Clelia Peano

[clelia.peano@itb.cnr.it](mailto:clelia.peano@itb.cnr.it)

Prof. Pietro Alifano

[pietro.alifano@unisalento.it](mailto:pietro.alifano@unisalento.it)

1. **Supplementary Figures**

**1.1 Supplementary Figure 1**

**
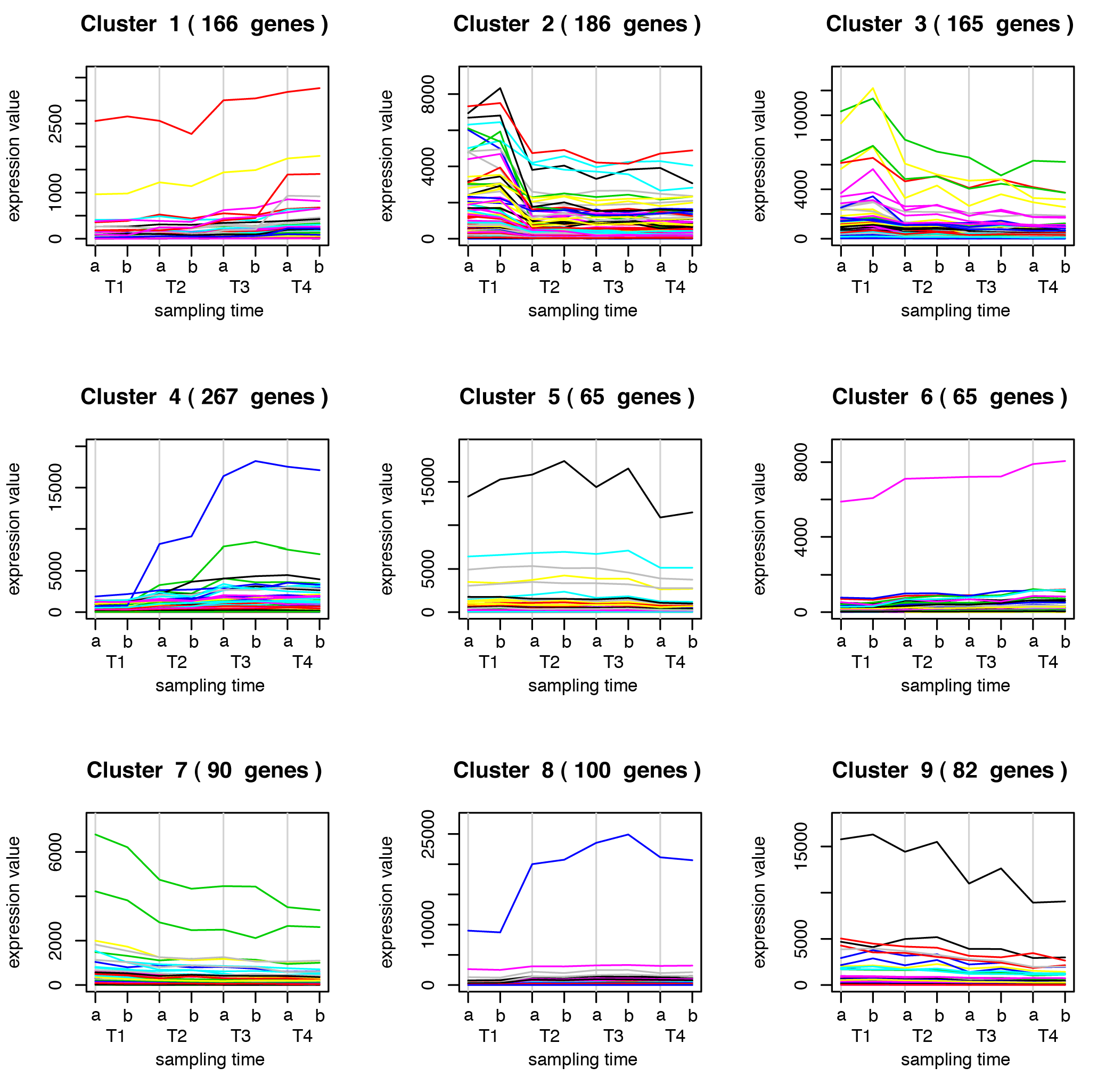
**

**Fig. S1.** maSigPro analysis of RNA-Seq time series dataset. The expression pattern of 1186 variable genes during the time course subdivided into 9 clusters is shown.

**1.2 Supplementary Figure 2**


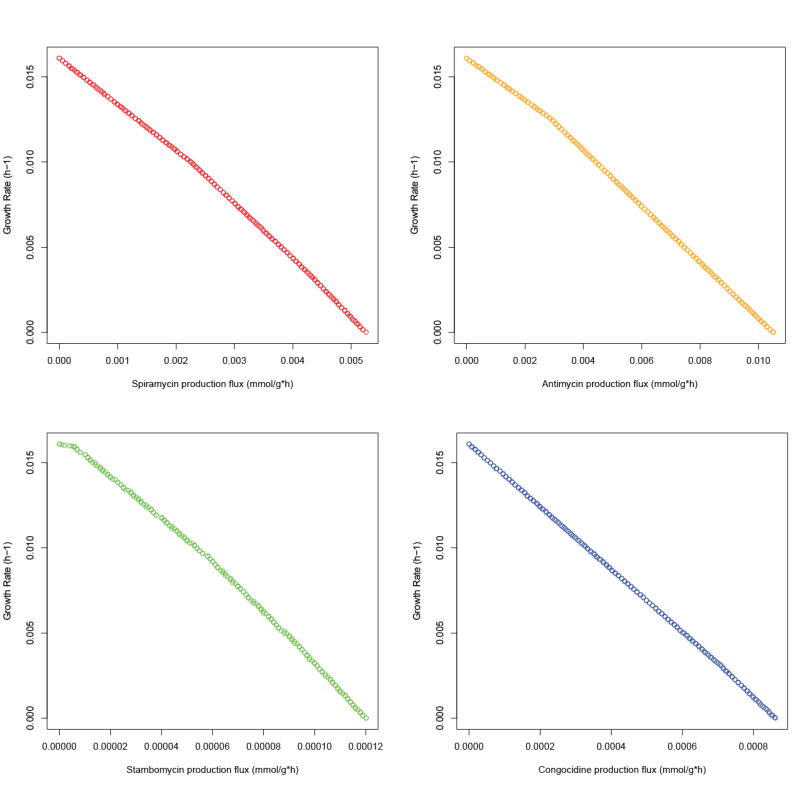


**Fig. S2.** Pareto front calculation between antibiotics production rates and growth rate.

**1.3 Supplementary Figure 3**


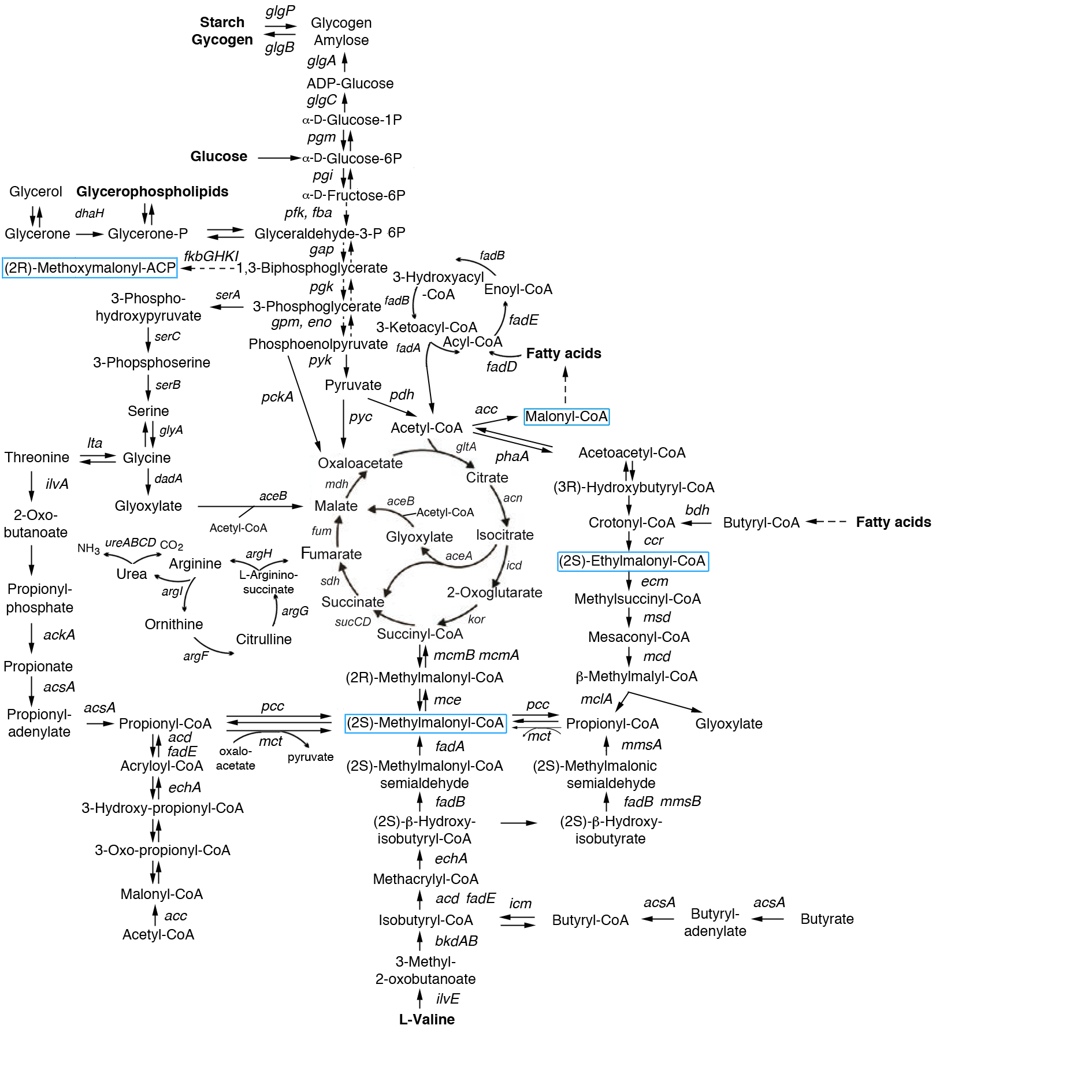


**Fig. S3.** Primary metabolic pathways feeding spiramycin biosynthesis. The spiramycin macrolactone backbone metabolic precursors malonyl-CoA, methylmalonyl-CoA, ethylmalonyl-CoA and methoxymalonyl-CoA are boxed in cyan.

**1.4 Supplementary Figure 4**

**
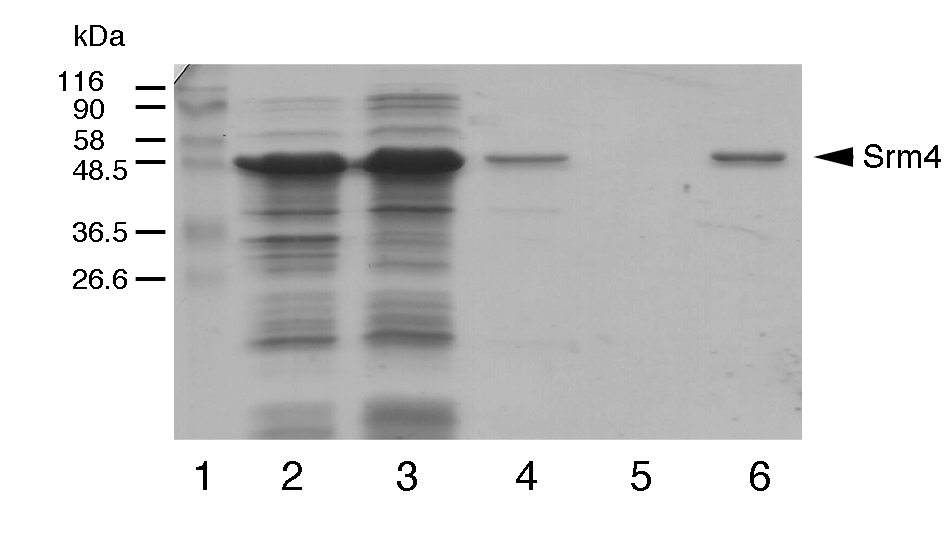
**

**Fig. S4.** Overproduction and purification of His_6_-tagged CCR (Srm4) protein. Different steps of His_6_-Srm4 overexpression in *E. coli* BL21 (DE3) and purification are shown: total protein extract from induced *E. coli* BL21 (DE3) cells harbouring pET/Srm4 plasmid (lane 2), soluble protein extract after cell lysis (lane 3), first affinity chromatography elution after soluble protein extract loading (pass through) (lane 4), affinity chromatography elution after washing steps (lane 5), affinity chromatography elution with purified His_6_-Srm4 protein (arrowhead). In lane 1, molecular weight ladder was loaded.

**1.5 Supplementary Figure 5**

**
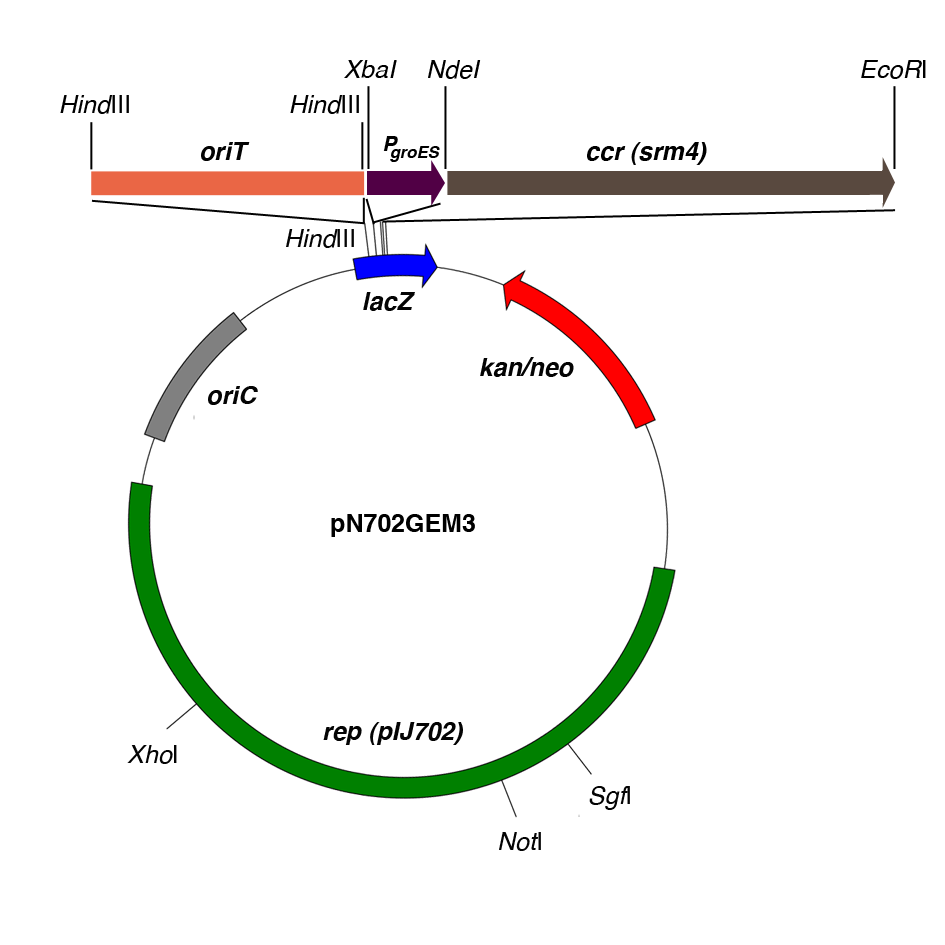
**

**Fig. S5.** Construction of recombinant plasmids. The *E. coli*-Streptomycetes shuttle vector pN702GEM3 was engineered by sequential insertion of: i.) the *oriT* sequence for conjugative transfer (generating pNGEM/OriT), ii.) the *P_groES_* region (generating pNGEM/OriT/P), and the *ccr* (*srm4*) gene (generating pNGEM/OriT/P/CCR).

**1.6 Supplementary Figure 6**

**
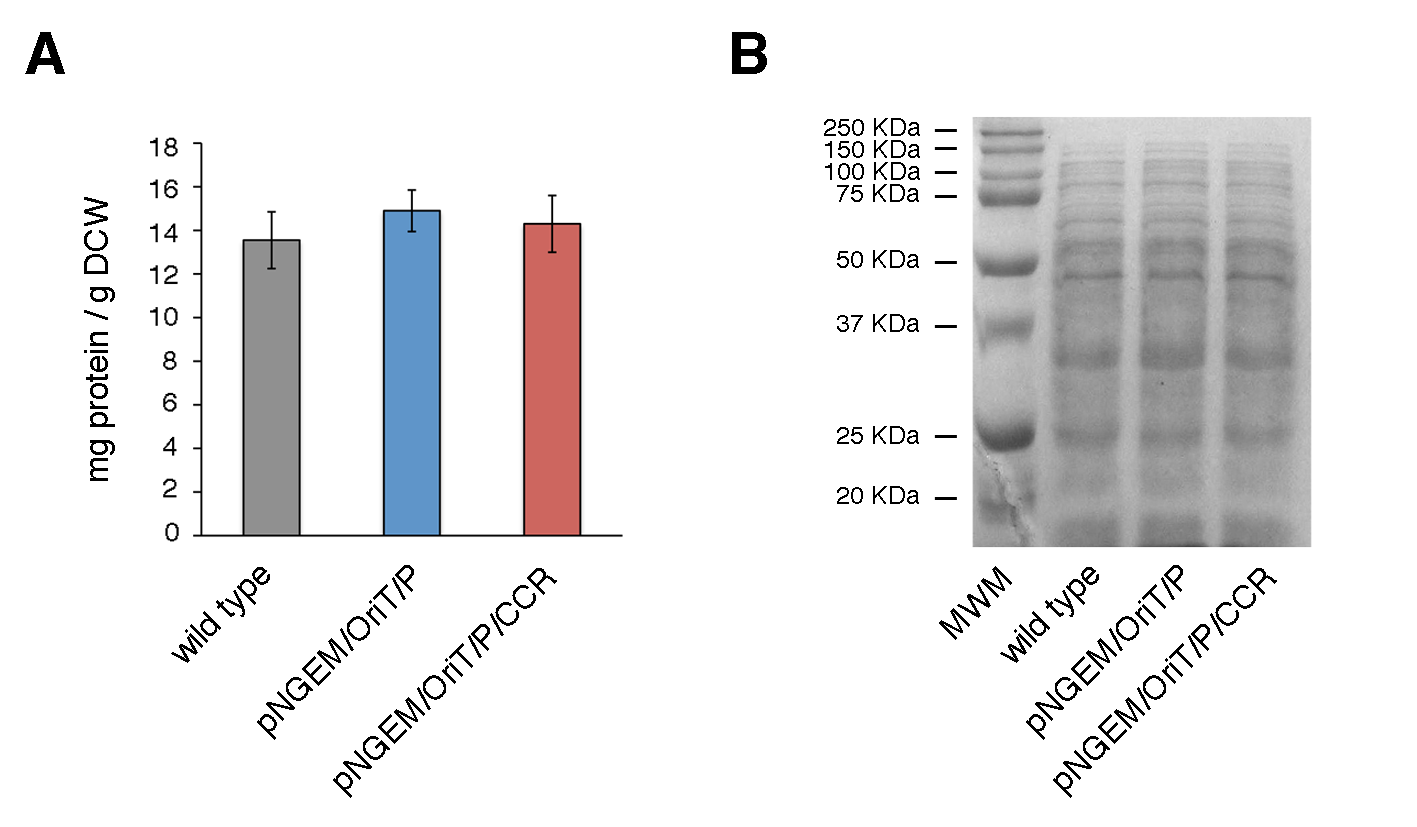
**

**Fig. S6.** Soluble protein content and electrophoretic pattern in wild type and recombinant strains. *S. ambofaciens* wild type strain and recombinant strains harbouring pNGEM/OriT/P or pNGEM/OriT/P/CCR plasmids were grown in YS broth for 48 h. Then bacteria were processed to obtain soluble protein extracts. Soluble protein content (expressed as mg protein/g DCW) and electrophoretic pattern were determined, respectively, by Bio-Rad colorimetric assays (A) and SDS-PAGE (B). In panel A, values represent means from three independent experiments. Bars represent standard deviations.

**2. Supplementary Tables**

**2.1 Table S1**

Read mapping, rRNA depletion, strand specificity and gene coverage evaluation for the analysed samples.

**2.2 Table S2**

C and N sources uptake rates were calculated from the corresponding original manuscript. Here we report the actual uptake rates used in the model when testing predicted growth rates against the set of available experimental results.

| **Lounès et al. 1996 (Glycerol + NH^4+^)** | | |
| --- | --- | --- |
| Compound | EX reaction in iMF1244 | Lower bound (mmol/g(CDW)*h |
| H_2_O | EX_cpd00001_e0 | -10 |
| H^+^ | EX_cpd00067_e0 | -10 |
| Mg^2+^ | EX_cpd00254_c0 | -10 |
| SO4^2-^ | EX_cpd00048_e0 | -10 |
| Zn^2+^ | EX_cpd00034_e0 | -10 |
| K^+^ | EX_cpd00205_e0 | -10 |
| PO4^2-^ | EX_cpd00009_e0 | -10 |
| Ca^2+^ | EX_cpd00063_e0 | -10 |
| Cl^-^ | EX_cpd00099_e0 | -10 |
| Na^+^ | EX_cpd00971_e0 | -10 |
| Co^2-^ | EX_cpd00149_e0 | -10 |
| Glycerol | EX_cpd00100_e0 | -1.1 |
| NH^4+^ | EX_cpd00013_e0 | -0.8 |
| O_2_ | EX_cpd00007_e0 | -10 |
| **Colombiè et al. 2005 (Glucose + Valine)** | | |
| Compound | EX reaction in iMF1244 | Lower bound (mmol/g(CDW)*h |
| H_2_O | EX_cpd00001_e0 | -10 |
| H^+^ | EX_cpd00067_e0 | -10 |
| Mg^2+^ | EX_cpd00254_c0 | -10 |
| SO4^2-^ | EX_cpd00048_e0 | -10 |
| Zn^2+^ | EX_cpd00034_e0 | -10 |
| K^+^ | EX_cpd00205_e0 | -10 |
| PO4^2-^ | EX_cpd00009_e0 | -10 |
| Ca^2+^ | EX_cpd00063_e0 | -10 |
| Cl^-^ | EX_cpd00099_e0 | -10 |
| Na^+^ | EX_cpd00971_e0 | -10 |
| Co^2-^ | EX_cpd00149_e0 | -10 |
| Glucose | EX_cpd00027_e0 | -0.8 |
| NH^4+^ | EX_cpd00013_e0 | -10 |
| O_2_ | EX_cpd00007_e0 | -10 |
| **Lounes et al. 1996 (Glucose+ Valine)** | | |
| Compound | EX reaction in iMF1244 | Lower bound (mmol/g(CDW)*h^-1^ |
| H_2_O | EX_cpd00001_e0 | -10 |
| H^+^ | EX_cpd00067_e0 | -10 |
| Mg^2+^ | EX_cpd00254_c0 | -10 |
| SO4^2-^ | EX_cpd00048_e0 | -10 |
| Zn^2+^ | EX_cpd00034_e0 | -10 |
| K^+^ | EX_cpd00205_e0 | -10 |
| PO4^2-^ | EX_cpd00009_e0 | -10 |
| Ca^2+^ | EX_cpd00063_e0 | -10 |
| Cl^-^ | EX_cpd00099_e0 | -10 |
| Na^+^ | EX_cpd00971_e0 | -10 |
| Co^2-^ | EX_cpd00149_e0 | -10 |
| Glucose | EX_cpd00027_e0 | -0.3 |
| Valine | EX_cpd00156_e0 | -10 |
| O_2_ | EX_cpd00007_e0 | -10 |
| **Lounes et al. 1995 (Glycerol+ Valine)** | | |
| Compound | EX reaction in iMF1244 | Lower bound (mmol/g(CDW)*h |
| H_2_O | EX_cpd00001_e0 | -10 |
| H^+^ | EX_cpd00067_e0 | -10 |
| Mg^2+^ | EX_cpd00254_c0 | -10 |
| SO4^2-^ | EX_cpd00048_e0 | -10 |
| Zn^2+^ | EX_cpd00034_e0 | -10 |
| K^+^ | EX_cpd00205_e0 | -10 |
| PO4^2-^ | EX_cpd00009_e0 | -10 |
| Ca^2+^ | EX_cpd00063_e0 | -10 |
| Cl^-^ | EX_cpd00099_e0 | -10 |
| Na^+^ | EX_cpd00971_e0 | -10 |
| Co^2-^ | EX_cpd00149_e0 | -10 |
| Glycerol | EX_cpd00100_e0 | -1.1 |
| Valine | EX_cpd00156_e0 | -1 |
| O_2_ | EX_cpd00007_e0 | -10 |

**2.3 Table S3**

iMAT gene expression data integration. 1st and 3rd quartiles for expression data at each time point used when mapping expressio data with iMAT.

| **Quartiles** | **Time point 1** | **Time point 2** | **Time point 3** | **Time point 4** |
| --- | --- | --- | --- | --- |
| 1^st^ | 10.07 | 9.32 | 9.90 | 11.14 |
| 3^rd^ | 120.07 | 124.60 | 125.61 | 130.37 |
| Flux activation threshold | 0.1 | 0.1 | 0.1 | 0.1 |

**2.4 Table S4**

Experimentally measured mycelial growth and spiramycin production rates. These values were used to constraint the metabolic reconstruction during growth simulations Genome-scale analysis of *Streptomyces coelicolor* A3(2) metabolism.

| **Time point** | **Time (hours)** | **Growth rate** | **Spiramycin production rate** |
| --- | --- | --- | --- |
| T1 | 48 | 0 | 1,73205E-05 |
| T2 | 72 | 0,003491062 | 0 |
| T3 | 96 | 0 | 4,94267E-06 |
| T4 | 120 | 0 | 0 |

**3 Supplementary files description**

**3.1 Data Sheet 1**

*S. ambofaciens* genome annotation integrated with RAST and antiSMASH results.

**3.2 Data Sheet 2**

Complete description of growth parameters and spiramycin production measured along the Time Course analysed

**3.3 Data Sheet 3**

MaSigPro analysis results

**3.4 Data Sheet 4**

Metabolic Model

**3.5 Data Sheet 5**

Complete list of the 65 reactions identified as key nodes for spiramycin production increase

**3.6 Data Sheet 6**

Spiramycin specific production rates (q_p_)
